# Altered expression of *SOCS* genes periodontitis

**DOI:** 10.1186/s12903-022-02602-7

**Published:** 2022-12-01

**Authors:** Soudeh Ghafouri-Fard, Leila Gholami, Saba Sadeghpour, Naghme Nazer, Bashdar Mahmud Hussen, Arezou Sayad, Mohammad Taheri

**Affiliations:** 1 Department of Medical Genetics, School of Medicine, Shahid Beheshti University of Medical Sciences, Tehran, Iran; 2 Department of Periodontics, Dental Research Center, Hamadan University of Medical Sciences, Hamadan, Iran; 3 Department of Electrical Engineering, Sharif University of Technology, Tehran, Iran; 4 Department of Pharmacognosy, College of Pharmacy, Hawler Medical University, Kurdistan Region, Erbil, Iraq; 5 Center of Research and Strategic Studies, Lebanese French University, Kurdistan Region, Erbil, Iraq; 6 Dental Research Center, Research Institute for Dental Sciences, Dental School, Shahid Beheshti University of Medical Sciences, Tehran, Iran; 7 Institute of Human Genetics, Jena University Hospital, Jena, Germany; 8 Urology and Nephrology Research Center, Shahid Beheshti University of Medical Sciences, Tehran, Iran

**Keywords:** SOCS, JAK/STAT, Periodontitis

## Abstract

Suppressor of cytokine signalling (SOCS) family comprises a group of proteins that impede JAK/STAT signalling, thus being involved in the pathogenesis of immune-related conditions. In the present work, we aimed at identification of the role of *SOCS* genes in the pathogenesis of periodontitis through evaluation of their expression levels both in the circulation and in the affected tissues of patients. Thus, we measured expression levels of *SOCS1-3* and *SOCS5* transcripts in the blood and gingival samples of patients with periodontitis in comparison with control samples obtained during dental crown lengthening. Expressions of *SOCS1, SOCS2, SOCS3* and *SOCS5* genes were similar between gingival tissues of patients and controls. However, our results demonstrated under-expression of *SOCS1* in blood samples of patients compared with controls (Ratio of mean expression (RME) = 0.47, *P* value = 0.04). The same pattern was observed among female subjects (RME = 0.38, *P* value = 0.04). *SOCS2* was down-regulated in blood samples of female patients compared with female controls (RME = 0.22, *P* value = 0.04). *SOCS3* was also under-expressed in the circulation of total cases versus total controls (RME = 0.29, *P* value = 0.02) and in female patients compared with female controls (RME = 0.19, *P* value = 0.04). Expression of *SOCS5* was not different between blood samples two study groups. *SOCS2* had the best function in separation of affected tissues from unaffected ones (AUC = 0.66, sensitivity = 0.39, specificity = 0.83). *SOCS3* was superior to other transcripts in differentiation of blood samples of patients from normal blood samples (AUC = 0.69, sensitivity = 0.81, specificity = 0.68). Combination of transcript levels of *SOCS1, SOCS2, SOCS3* and *SOCS5* genes enhanced the AUC values to 0.64 and 0.67 in tissue and blood specimens, respectively. Taken together, certain *SOCS* genes have been found to be dysregulated in the circulation of patients with periodontitis.

## Introduction

Periodontal disorders impose an important burden on health [1]. The resident flora of the oral cavity contributes in the pathogenesis of periodontal disorders. Some central features of the oral microbiome have been clarified using metagenomic and 16S gene analyses [2]. These techniques have also facilitated establishment of personalized medicine approaches that incorporates genomic and clinical data to forecast possible predisposition to these disorders [3]. Thus, expression assays in tissues obtained from patients with periodontitis have practical significance. Suppressor of cytokine signalling (SOCS) family comprises a group of molecules that inhibit JAK/STAT signals [4]. JAK/STAT signalling is a cytokine-induced pathway that regulates various critical biological responses, particularly immune function [4]. This family consists of eight proteins, namely CIS and SOCS1-SOCS7, all of them having a SH2 domain, SOCS box, and a distinctive N-terminal part [5]. SOCS1-3 and CIS are proteins whose expressions are stimulated by cytokines or growth factors, leading to suppression of JAK/STAT-associated cytokine signalling through a negative feedback circuit [4]. SOCS5 is another member of this family that contain SOCS box, yet the biological effects of this protein are not completely understood. Expression of this protein has been detected in primary B and T lymphoid cells, yet it does not exert essential function in production and activity of these cells [6].

Consistent with the important roles of SOCS proteins in the modulation of inflammatory responses, these proteins participate in the pathogenesis of related disorders. Periodontitis is among complex disorders in which inflammatory responses regulate disease progression [7]. A number of previous studies have indicated the role of SOCS proteins in this disorder. For instance, expression of SOCS1 has been found to be increased in the experimental model of periodontitis, parallel with activation of STAT1 and NF-κB pathways [8]. Moreover, down-regulation of SOCS3 in myeloid cells has resulted in enhancement of *Porphyromonas gingivalis* -induced inflammatory responses and over-production of interleukins-1β, -6, and -8 by peritoneal macrophages [9].

SOCS proteins have a crucial role in the regulation of microbial pathogen-associated signaling of cytokines, mainly via suppression of the activity of JAK/STAT cascade. In addition, they are involved in various processes exploited by bacteria to evade host defense [10].

SOCS1-3 have also been suggested to contribute in the regulation of bone resorption in the context of periodontitis [11]. Therefore, assessment of expression of these genes might facilitate identification of the underlying cause of periodontitis and pathogenic events associated this disorder.

In this work, we aimed at identification of the role of *SOCS* genes in the pathogenesis of periodontitis through evaluation of their expression levels both in the circulation and in the affected tissues of patients. Thus, we measured expression levels of *SOCS1-3* and *SOCS5* transcripts in the circulation and gingiva of patients with periodontitis in comparison with control samples obtained during dental crown lengthening.

## Materials and methods

### Enlisted persons

Whole blood samples were obtained from patients with periodontitis and control subjects. Gingival samples were obtained from individuals affected with chronic periodontitis, similar to the method explained in our former study [12]. Inclusion criteria were chronic periodontitis (Stage II to IV), probing depth of 5 mm or greater, bleeding on probing, and a minimum of 3 mm attachment loss requiring surgical periodontal management. We excluded individuals who had history of smoking, systemic disorders, diabetes mellitus and consumption of antibiotics or anti-inflammatory agents. All patients were assessed in a university-affiliated periodontal clinic. Control samples were excised during dental crown lengthening procedure. Ethical committee of Shahid Beheshti University of Medical Sciences permitted the conduction of study based on the ethical guidelines (Ethical code IR.SBMU.DRC.REC.1400.012).

### Assessment of gene expression

PicoPure™ RNA Isolation Kit (Applied Biosystems) was used for RNA extraction. Then, cDNA was produced from these samples by using the cDNA production kit (Yektatajhiz, Iran). Expressions of *SOCS1-3* and *SOCS5* genes were measured in tissues and blood samples using the qRT-PCR kit (Ampliqon, Denmark) in LightCycler® 96 machine. Reactions were performed in duplicate. Details about PCR conditions and primers are reported in our former study [13].

### Statistical methods

Data was assessed using R programming language (pROC, qreg, and Stan and loo packages). Expression of *SOCS1-3* and *SOCS5* genes was calculated using Ct and efficiency. Values were log-transformed. The normality of data was assessed using Shapiro–Wilk test. Since data was non-parametric, Mann–Whitney U test was used for comparison of mean values. Spearman correlation coefficient was measured to estimate correlation between expressions of *SOCS1-3* and *SOCS5* genes. Diagnostic power of these genes was assessed through depicting receiver operating characteristic curves using pROC-package. The level of statistical significance was set at *P* < 0.05.

## Results

### General data

A total of 26 patients with periodontitis (Female/male ratio: 16/10) and 28 controls (female/male ratio: 12/16) were enrolled. Cases and controls were matched in terms of their age (Mean age ± SD: 37.6 ± 2.5 versus 37.5 ± 1.7).

### Experiments

**Fig. 1:**
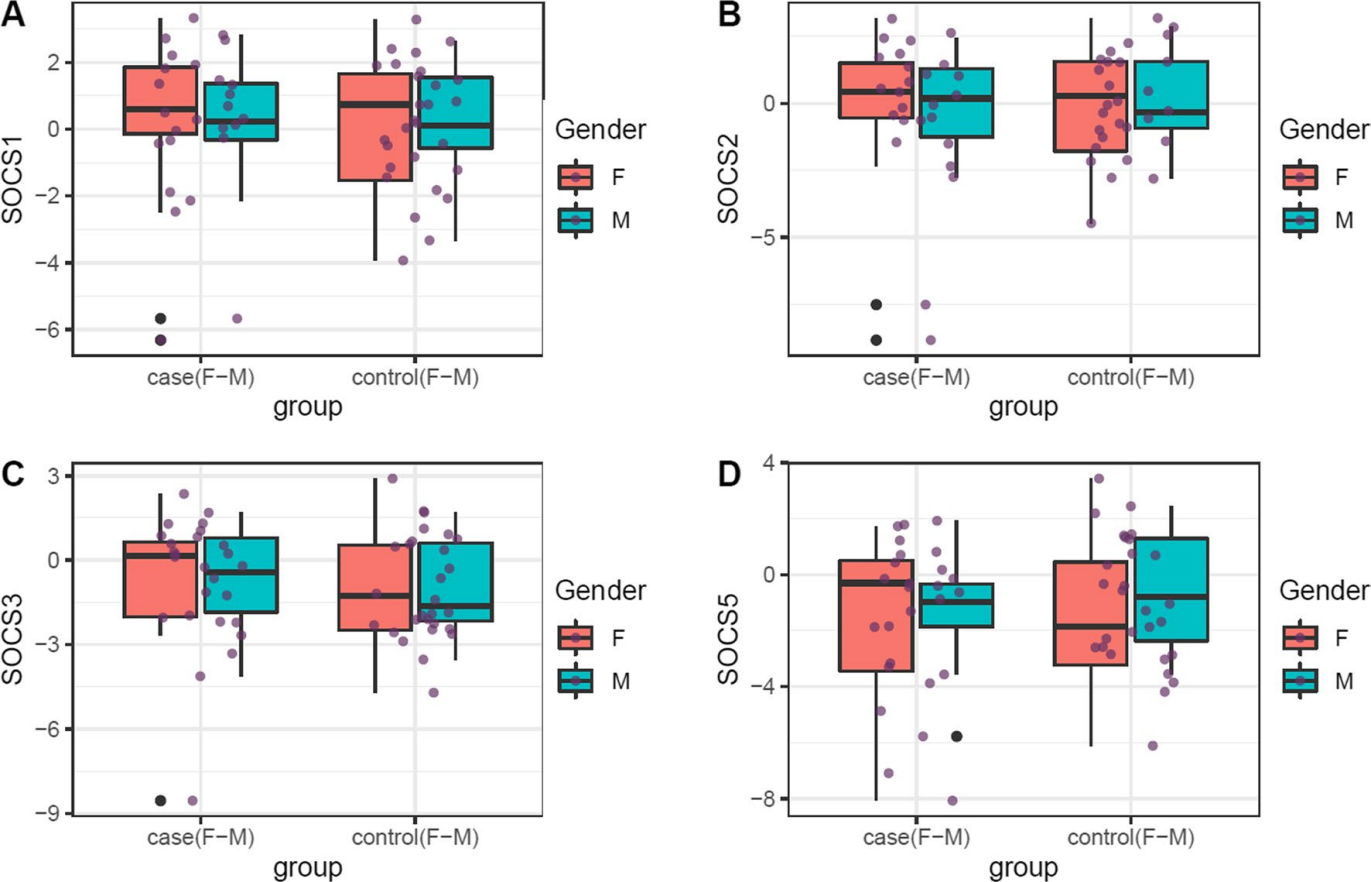
Expression of *SOCS1, SOCS2, SOCS3* and *SOCS5* genes in tissues of patients compared with controls. All data points from maximum to minimum are shown in box plots and whiskers. Boxes are illustrated from Q1 to Q3. The horizontal lines in the middle of boxes represent the median values. Each black dot represents transcript level in an individual sample. Mean values and interquartile range are shown

**Fig. 2:**
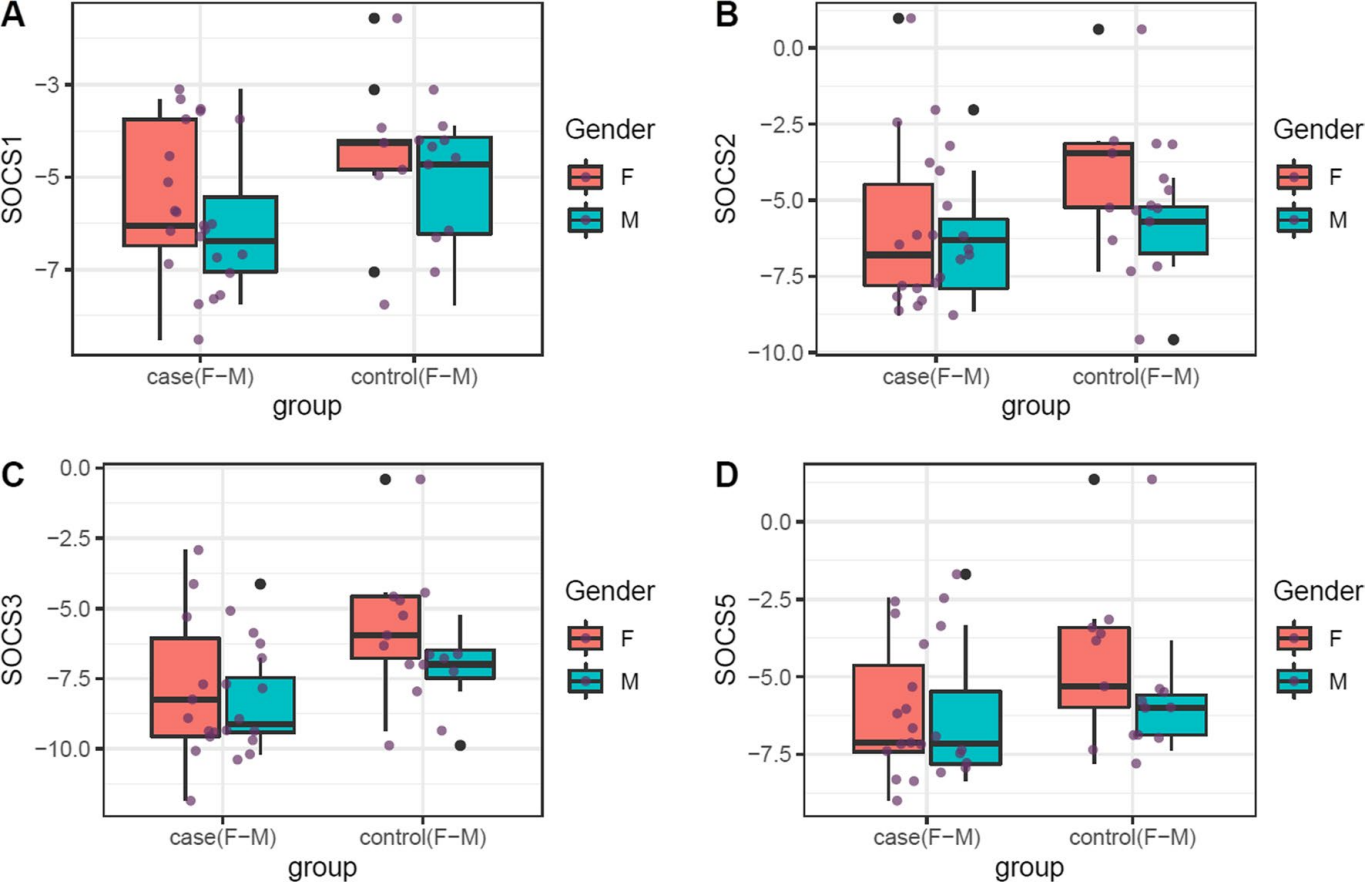
Expression of *SOCS1, SOCS2, SOCS3* and *SOCS5* genes in blood of patients versus controls. All data points from maximum to minimum are shown in box plots and whiskers. Boxes are illustrated from Q1 to Q3. The horizontal lines in the middle of boxes represent the median values. Each black dot represents transcript level in an individual sample. Mean values and interquartile range are shown

Expressions of *SOCS1, SOCS2, SOCS3* and *SOCS5* genes in tissue and blood of study subgroups are shown in Figs. 1 and 2, respectively.

Expressions of *SOCS1, SOCS2, SOCS3* and *SOCS5* genes were similar between gingival tissues of patients and controls. However, expression of *SOCS1* was lower in blood samples of patients in comparison with unaffected individuals (Ratio of mean expression (RME) = 0.47, *P* value = 0.04). The same pattern was observed among female subjects (RME = 0.38, *P* value = 0.04). *SOCS2* was under-expressed in blood samples of female patients compared with female controls (RME = 0.22, *P* value = 0.04). *SOCS3* was also under-expressed in the circulation of total patients compared with total controls (RME = 0.29, *P* value = 0.02) and in female patients compared with female controls (RME = 0.19, *P* value = 0.04). Blood levels of *SOCS5* were not changed between subgroups (Table 1).

**Table 1:** Assessment of expression of *SOCS1, SOCS2, SOCS3* and *SOCS5* genes in tissues and blood specimens of cases versus controls

Number of Samples		*SOCS1*					*SOCS2*					*SOCS3*					*SOCS5*				
		SE	RME	*P* value	95% CI		SE	RME	*P* value	95% CI		SE	RME	*P* value	95% CI		SE	RME	*P* value	95% CI	
*Tissues*																					
Total	26/28	0.58	1.05	0.91	− 1.10	1.24	0.66	0.88	0.78	− 1.50	1.14	0.57	1.20	0.65	− 0.88	1.40	0.69	0.71	0.47	− 1.89	0.88
F	16/12	0.93	1.01	0.99	− 1.89	1.91	1.05	0.83	0.80	− 2.42	1.89	0.89	1.18	0.79	− 1.60	2.07	1.11	0.85	0.84	− 2.51	2.05
M	10/16	0.68	1.16	0.76	− 1.21	1.63	0.70	1.04	0.93	− 1.39	1.52	0.74	1.21	0.71	− 1.28	1.83	0.87	0.68	0.53	− 2.39	1.29
*Blood*																					
Total	23/17	0.50	0.47	0.04	− 2.11	− 0.08	0.76	0.42	0.11	− 2.80	0.30	0.72	0.29	0.02	− 3.25	− 0.32	0.70	0.50	0.16	− 2.43	0.41
F	15/10	0.63	0.38	0.04	− 2.71	− 0.09	0.97	0.22	0.04	− 4.19	− 0.17	1.04	0.19	0.04	− 4.56	− 0.17	0.98	0.33	0.12	− 3.67	0.47
M	8/7	0.79	0.60	0.37	− 2.43	0.96	1.03	1.02	0.97	− 2.18	2.25	0.89	0.47	0.24	− 3.04	0.84	0.96	0.88	0.85	− 2.31	1.94

**Fig. 3:**
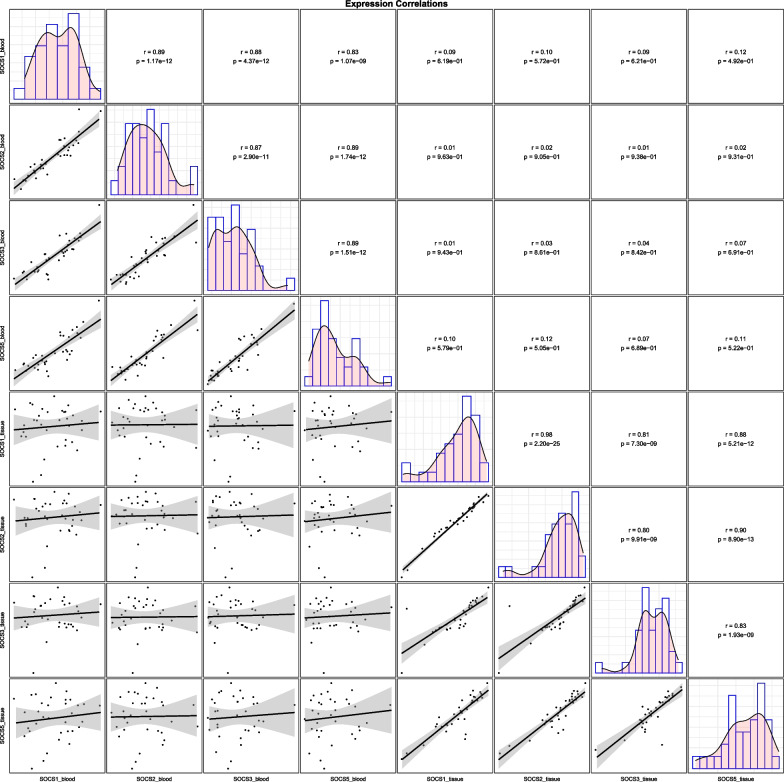
Correlations between tissue and blood expressions of *SOCS1, SOCS2, SOCS3* and *SOCS5* genes. Distribution of expression data is signified on the diagonals. A bivariate scatter plot with a fitted line is depicted for each correlation on the inferior part of the diagonals. Correlation coefficients and P values are mentioned on the upper parts

While expressions of *SOCS* genes were strictly correlated in each set of samples, no correlation was found between their tissue and blood levels (Fig. 3).

**Fig. 4:**
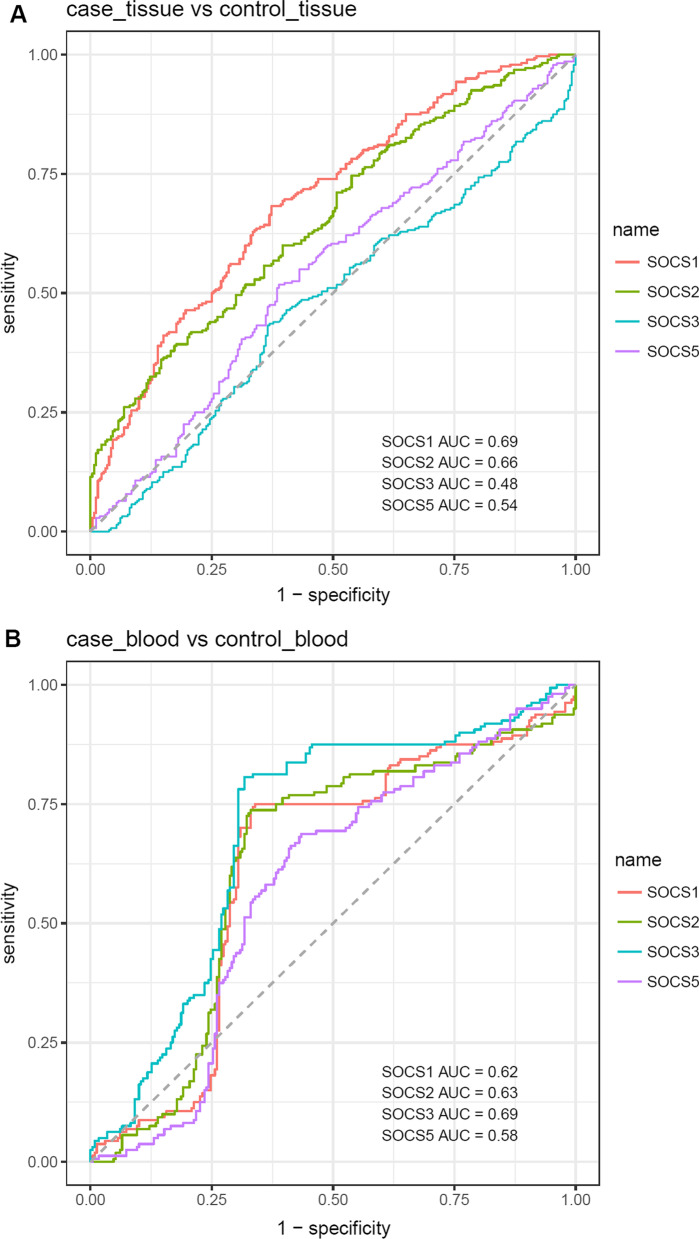
Bayesian generalized linear model-based ROC curves

Finally, we measured the diagnostic power of *SOCS* genes in blood and tissue samples using the Bayesian Generalized Linear Model (Fig. 4).

*SOCS2* had the best performance in separation of affected tissues from unaffected tissue samples (AUC = 0.66, sensitivity = 0.39, specificity = 0.83). *SOCS3* was superior to other transcripts in differentiation of blood samples of cases from normal blood samples (AUC = 0.69, sensitivity = 0.81, specificity = 0.68). Combination of transcript levels of *SOCS1, SOCS2, SOCS3* and *SOCS5* genes enhanced the AUC values to 0.64 and 0.67 in tissue and blood samples, respectively (Table 2).

**Table 2:** ROC curves-related statistics in tissue and blood samples

Samples	*SOCS1*			*SOCS2*			*SOCS3*			*SOCS5*			All		
	AUC	Sensitivity	Specificity	AUC	Sensitivity	Specificity	AUC	Sensitivity	Specificity	AUC	Sensitivity	Specificity	AUC	Sensitivity	Specificity
Tissue	0.69	0.68	0.63	0.66	0.39	0.83	0.48	0.43	0.63	0.54	0.52	0.61	0.64	0.71	0.52
Blood	0.62	0.75	0.66	0.63	0.74	0.67	0.69	0.81	0.68	0.58	0.69	0.57	0.67	0.78	0.65

## Discussion

Periodontitis is a disorder with high burden on personal health. Metagenomics and periodontal flap design have been found to be risk factors of developing periodontitis [1, 2]. In an attempt to find some aspects of immune dysregulation in periodontitis, we assessed expression levels of *SOCS* genes in the blood and gingival tissues of periodontitis patients compared with matched controls. We found similar levels of *SOCS* genes between gingival tissues of patients and controls. Yet, we found under-expression of *SOCS1* in blood samples of patients compared with controls. *SOCS2* and *SOCS3* were down-regulated in blood samples of female patients compared with female controls. *SOCS3* was also under-expressed in the circulation of total cases versus total controls. Expression of *SOCS5* was not different between blood samples two study groups.

SOCS1 has been firstly recognized as a negative modulator of the JAK/STAT-mediated signaling pathway induced by IL-4, IL-6, and IFN-γ cytokines [14–16]. Several cytokines have been dysregulated in the course of periodontitis contributing in the pathoetiology of this condition. Most importantly, release of IL-6 and TNF family cytokines by periodontal and immune cells following stimulation by pathogens induces and attracts specific immune cells causing tissue injury [17]. Release of IL-4 by naive CD4 + T cells and B cells has a role in activation of STAT6 and GATA3 and stimulation of differentiation of Th2 cells and B cells [18, 19]. A previous meta-analysis has indicated consistent down-regulation of IL-4 in patients with chronic periodontitis and elevation of its levels following treatment of this condition [20]. On the other hand, gingival crevicular fluid levels of IL-6 and IFN-γ have been found to be remarkably higher in these patients compared with healthy controls [20]. Another study has detected parallel up-regulation of SOCS1 and IFN-γ in the experimental model of periodontitis [8]. These findings indicate the presence of a complicated network between cytokines, their regulatory factors and their mediators in the context of periodontitis. Down-regulation of SOCS1 in the blood samples of patients with periodontitis might lead to up-regulation of several immune-related pathways and molecules which are regulated by this protein. The consequent up-regulation of activity of these pathways might affect the pathogenesis of periodontitis in the gingival tissues.

*SOCS2* was under-expressed in blood samples of female patients compared with female control group. This member of SOCS protein family has a fundamental role in the regulation of balance between immune responses and oxidative stress [21]. Thus, dysregulation of this protein might affect the regenerative processes in the course of periodontitis.

*SOCS3* was also under-expressed in the circulation of total patients versus total control group and in female patients compared with female control group. SOCS3 is regarded as an important negative regulator of alveolar bone loss in periodontitis [9]. Thus, down-regulation of SOCS3 might lead to increased alveolar bone loss in these patients.

The observed sex-biased alterations in the expressions of *SOCS* genes in the current study might be due to small number of male subjects in the current study. Alternatively, *SOCS* genes might exert a sex-based role in the pathogenesis of periodontitis.

The results of the current study are in line with other studies reporting abnormal expressions of immune-related genes in the blood of patients with periodontitis. For instance, two NF-κB-related genes, namely DILC and FBXL19-AS have been shown to be down-regulated both in blood samples of these patients compared with control samples [22]. A number of other long non-coding RNAs, namely Linc0116, Linc00667, CDK6-AS1, FENDRR and DIRC3 have been found to be down-regulated in the blood samples of patients with periodontitis [23].

Finally, we appraised diagnostic power of *SOCS* transcripts in the context of periodontitis. *SOCS2* had the best performance in separation of affected tissues from unaffected ones. *SOCS3* was superior to other transcripts in differentiation of blood samples of patients from normal blood samples. Integration of transcript levels of *SOCS1, SOCS2, SOCS3* and *SOCS5* genes improved the AUC values in both tissue and blood samples.

Taken together, *SOCS*1, *SOCS2* and *SOCS3* genes have been found to be dysregulated in the circulation of patients with periodontitis. These transcripts might be regarded as peripheral markers for periodontitis. Yet, tissue levels of these transcripts were similar between two study subgroups, indicating that these transcripts possibly regulate systemic but not local immune responses in this condition. Our study has a limitation regarding the sample size. Thus, we propose conduction of further studies with larger sample sizes for verification of the results of the current study. Particularly, the obtained AUC values should be verified in independent studies. Future in vitro studies are also needed to evaluate the function of *SOCS* genes in the pathophysiology of periodontitis.

## Data Availability

All data generated or analysed during this study are included in this published article.

## References

[1] Briguglio F, Zenobio EG, Isola G, Briguglio R, Briguglio E, Farronato D (2011). Complications in surgical removal of impacted mandibular third molars in relation to flap design: clinical and statistical evaluations. Quintessence Int.

[2] Martellacci L, Quaranta G, Patini R, Isola G, Gallenzi P, Masucci L (2019). A Literature review of metagenomics and culturomics of the peri-implant microbiome: current evidence and future perspectives. Materials.

[3] Matarese G, Ramaglia L, Fiorillo L, Cervino G, Lauritano F, Isola G (2017). Implantology and periodontal disease: the panacea to problem solving?. Open Dent J.

[4] Cooney RN (2002). Suppressors of cytokine signaling (SOCS): inhibitors of the JAK/STAT pathway. Shock.

[5] Krebs DL, Hilton DJ (2000). SOCS: physiological suppressors of cytokine signaling. J Cell Sci.

[6] Brender C, Columbus R, Metcalf D, Handman E, Starr R, Huntington N (2004). SOCS5 is expressed in primary B and T lymphoid cells but is dispensable for lymphocyte production and function. Mol Cell Biol.

[7] Cekici A, Kantarci A, Hasturk H (2000). Van Dyke TE 2014 Inflammatory and immune pathways in the pathogenesis of periodontal disease. Periodontol.

[8] de Souza JAC, Nogueira AVB, de Souza PPC, de Oliveira GJPL, de Medeiros MC, Garlet GP (2017). Suppressor of cytokine signaling 1 expression during LPS-induced inflammation and bone loss in rats. Braz Oral Res.

[9] Papathanasiou E, Kantarci A, Konstantinidis A, Gao H, Van Dyke TE (2016). SOCS-3 regulates alveolar bone loss in experimental periodontitis. J Dent Res.

[10] Duncan SA, Baganizi DR, Sahu R, Singh SR, Dennis VA (2017). SOCS proteins as regulators of inflammatory responses induced by bacterial infections: a review. Front Microbiol.

[11] Santos MRG, Queiroz-Junior CM, Madeira MFM, Machado FS (2020). Suppressors of cytokine signaling (SOCS) proteins in inflammatory bone disorders. Bone.

[12] Gholami L, Ghafouri-Fard S, Mirzajani S, Arsang-Jang S, Taheri M, Dehbani Z (2020). The lncRNA ANRIL is down-regulated in peripheral blood of patients with periodontitis. Noncoding RNA Res.

[13] Ghafouri-Fard S, Oskooei VK, Azari I, Taheri M (2018). Suppressor of cytokine signaling (SOCS) genes are downregulated in breast cancer. World J Surg Oncol.

[14] Starr R, Willson TA, Viney EM, Murray LJ, Rayner JR, Jenkins BJ (1997). A family of cytokine-inducible inhibitors of signalling. Nature.

[15] Endo TA, Masuhara M, Yokouchi M, Suzuki R, Sakamoto H, Mitsui K (1997). A new protein containing an SH2 domain that inhibits JAK kinases. Nature.

[16] Naka T, Narazaki M, Hirata M, Matsumoto T, Minamoto S, Aono A (1997). Structure and function of a new STAT-induced STAT inhibitor. Nature.

[17] Pan W, Wang Q, Chen Q (2019). The cytokine network involved in the host immune response to periodontitis. Int J Oral Sci.

[18] Le Gros G, Ben-Sasson SZ, Seder R, Finkelman FD, Paul WE (1990). Generation of interleukin 4 (IL-4)-producing cells in vivo and in vitro: IL-2 and IL-4 are required for in vitro generation of IL-4-producing cells. J Exp Med.

[19] Vitetta ES, Ohara J, Myers CD, Layton JE, Krammer PH, Paul WE (1985). Serological, biochemical, and functional identity of B cell-stimulatory factor 1 and B cell differentiation factor for IgG1. J Exp Med.

[20] Stadler AF, Angst PD, Arce RM, Gomes SC, Oppermann RV, Susin C (2016). Gingival crevicular fluid levels of cytokines/chemokines in chronic periodontitis: a meta-analysis. J Clin Periodontol.

[21] Monti-Rocha R, Cramer A, GaioLeite P, Antunes MM, Pereira RVS, Barroso A (2019). SOCS2 is critical for the balancing of immune response and oxidate stress protecting against acetaminophen-induced acute liver injury. Front Immunol.

[22] Ghafouri-Fard S, Gholami L, Nazer N, Hussen BM, Shadnoush M, Sayad A (2022). Assessment of expression of NF-κB-related genes in periodontitis. Gene Rep.

[23] Ghafouri-Fard S, Gholami L, Badrlou E, Sadeghpour S, Nazer N, Shadnoush M (2021). Dysregulation of lncRNAs in circulation of patients with periodontitis: results of a pilot study. BMC Oral Health.

